# 

**Published:** 1875-06

**Authors:** 


### CONTENTS:

ORIGINAL COMMUNICATIONS:

ART. I. Examination of Brain. By Jos. G. Richardson, M.D., Philadelphia .... 401

ART. II. Hereditary Transmission and Variation by Descent .... 403

PROGRESS IN MEDICAL SCIENCES:

ART. I. Progress of Surgery. By Jno. E. Owens, M.D., Chicago .... 412

HOSPITALS:

St. Joseph's—Service of Prof. Gunn .... 421

CLINICS:

Ill. Charitable Eye and Ear Infirmary—Dr. Holmes .... 427

SELECTIONS.

The Neurotic Origin of Disease, etc.—Dr. Lente .... 431

EDITORS' BOOK TABLE .... 460

Institution for Feeble-Minded .... 476

MORTALITY STATISTICS, Chicago .... 479

